# Supplementary material for: Stimulation of Piezo1 by mechanical signals promotes bone anabolism
Source: eLife. 2019 Oct 7;8:e49631. doi: 10.7554/eLife.49631 (PMC6779475; doi:10.7554/eLife.49631)
Supplement: Transparent reporting form [file elife-49631-transrepform.pdf]

eLIFE
1st Floor
24 Hills Road
Cambridge CB2 1JP, UK
P 01223 855340
W elifesciences.org
T @elife

eLife's transparent reporting form

We encourage authors to provide detailed information *within their submission* to facilitate the interpretation and replication of experiments. Authors can upload supporting documentation to indicate the use of appropriate reporting guidelines for health-related research (see EQUATOR Network), life science research (see the BioSharing Information Resource), or the ARRIVE guidelines for reporting work involving animal research. Where applicable, authors should refer to any relevant reporting standards documents in this form.

If you have any questions, please consult our Journal Policies and/or contact us: editorial@elifesciences.org.

Sample-size estimation

- You should state whether an appropriate sample size was computed when the study was being designed
- You should state the statistical method of sample size computation and any required assumptions
- If no explicit power analysis was used, you should describe how you decided what sample (replicate) size (number) to use

Please outline where this information can be found within the submission (e.g., sections or figure legends), or explain why this information doesn't apply to your submission:

There are no previous experiments to use for estimation of the expected differences for Piezo1 deletion or Yoda1 administration in bone. Based on our previous studies with other genes in bone, the standard deviation for osteoblast surface per bone surface is 0.05 in wild type mice. We would be interested in any differences between genotypes greater than 0.07. Assuming equal variance in the two groups, a two-tailed t-test, an alpha of 0.05, and power of 0.80, we determined that we would need about 10 animals per group to detect an effect as small as 0.07. For anabolic loading experiment, we performed a pilot experiment to determine the effect size of anabolic loading on bone and found that 6 mice per groups was sufficient to detect an significant increase in bone formation induced by compressive loading.

Replicates

- You should report how often each experiment was performed
- You should include a definition of biological versus technical replication
- The data obtained should be provided and sufficient information should be provided to indicate the number of independent biological and/or technical replicates
- If you encountered any outliers, you should describe how these were handled
- Criteria for exclusion/inclusion of data should be clearly stated
- High-throughput sequence data should be uploaded before submission, with a private link for reviewers provided (these are available from both GEO and ArrayExpress)

Please outline where this information can be found within the submission (e.g., sections or figure legends), or explain why this information doesn't apply to your submission:

eLife Sciences Publications, Ltd is a limited liability non-profit non-stock corporation incorporated in the State of Delaware, USA, with company number 5030732, and is registered in the UK with company number FC030576 and branch number BR015634 at the address 1st Floor, 24 Hills Road, Cambridge CB2 1JP | August 2014

All in vitro cell culture experiments were performed three times with three technical replicates. Ex vivo organ cultures were performed three times with three biological replicates. This information was indicated in the methods section. For in vivo experiments, we analyzed the skeletal phenotype of conditional knockout mice at different ages and obtained similar results. For compressive loading experiment, we obtained an increase in bone formation in control mice similar to what we observed in our pilot experiment and similar to what other labs observed. No data were excluded. If bone samples were broken during tissue harvest, they were not used for further analyses.

Statistical reporting

- Statistical analysis methods should be described and justified
- Raw data should be presented in figures whenever informative to do so (typically when N per group is less than 10)
- For each experiment, you should identify the statistical tests used, exact values of N, definitions of center, methods of multiple test correction, and dispersion and precision measures (e.g., mean, median, SD, SEM, confidence intervals; and, for the major substantive results, a measure of effect size (e.g., Pearson's r, Cohen's d)
- Report exact p-values wherever possible alongside the summary statistics and 95% confidence intervals. These should be reported for all key questions and not only when the p-value is less than 0.05.

Please outline where this information can be found within the submission (e.g., sections or figure legends), or explain why this information doesn't apply to your submission:

Mean ± s.d. was used in our study. Exact sample number for each experiment was provided in figure legends. Two-way ANOVA analysis was used to detect statistically significant treatment effects among different genotypes (cells and mice). Two-tailed t-test was used to detect statistical difference between genotypes. This information was indicated in the figure legends or methods section.

(For large datasets, or papers with a very large number of statistical tests, you may upload a single table file with tests, Ns, etc., with reference to sections in the manuscript.)

Group allocation

- Indicate how samples were allocated into experimental groups (in the case of clinical studies, please specify allocation to treatment method); if randomization was used, please also state if restricted randomization was applied
- Indicate if masking was used during group allocation, data collection and/or data analysis

Please outline where this information can be found within the submission (e.g., sections or figure legends), or explain why this information doesn't apply to your submission:

For basal skeletal phenotype analysis and compressive loading experiment, mice were grouped based on their genotype. For Yoda1 administration, mice were assigned to Veh or Yoda1 groups based on their body weight. Specifically, mice were rank-ordered by body weight and then assigned the number 1 or 2, successively. Animals with the same number were assigned to the same operation group to give identical group means. All mice, and samples from those mice, were assigned an experimental identification number. All individuals involved in sample analyses had access only to the identification number, not the animal genotype or experimental group. Thus all investigators involved in data collection were blinded as to the genotype and group of the mice or samples. This information was indicated in the methods section.

Additional data files ("source data")

- We encourage you to upload relevant additional data files, such as numerical data that are represented as a graph in a figure, or as a summary table
- Where provided, these should be in the most useful format, and they can be uploaded as "Source data" files linked to a main figure or table
- Include model definition files including the full list of parameters used
- Include code used for data analysis (e.g., R, MatLab)

- Avoid stating that data files are "available upon request"

Please indicate the figures or tables for which source data files have been provided:

RNAseq source data for Figure 1A and supplementary Figure 1 has been deposited in BioProject under accession code PRJNA551282.
